# Immune System Alterations and Postpartum Mental Illness: Evidence From Basic and Clinical Research

**DOI:** 10.3389/fgwh.2021.758748

**Published:** 2022-02-10

**Authors:** Courtney Dye, Kathryn M. Lenz, Benedetta Leuner

**Affiliations:** ^1^Neuroscience Graduate Program, The Ohio State University, Columbus, OH, United States; ^2^Department of Psychology, The Ohio State University, Columbus, OH, United States; ^3^Department of Neuroscience, The Ohio State University, Columbus, OH, United States; ^4^Institute of Behavioral Medicine Research, The Ohio State University, Columbus, OH, United States

**Keywords:** cytokines, inflammation, microglia, postpartum depression, postpartum anxiety, postpartum psychosis

## Abstract

The postpartum period is a time associated with high rates of depression and anxiety as well as greater risk for psychosis in some women. A growing number of studies point to aberrations in immune system function as contributing to postpartum mental illness. Here we review evidence from both clinical and animal models suggesting an immune component to postpartum depression, postpartum anxiety, and postpartum psychosis. Thus far, clinical data primarily highlights changes in peripheral cytokine signaling in disease etiology, while animal models have begun to provide insight into the immune environment of the maternal brain and how central inflammation may also be contributing to postpartum mental illnesses. Further research investigating peripheral and central immune function, along with neural and endocrine interactions, will be important in successfully developing novel prevention and treatment strategies for these serious disorders that impact a large portion of new mothers.

## Introduction

A vast majority of women will experience pregnancy at some point during their lives (1). Pregnancy is a process that affects nearly every bodily system (2). The widespread and dramatic physiological changes of pregnancy occur to facilitate the successful development and, later, care of the offspring. However, these modifications also introduce a period of vulnerability to mental illness for the mother (Table 1). While the majority (~85%) of new mothers will experience a transient mood disruption early in the postpartum period known as the “baby blues,” ~10–20% of all parturient women will develop postpartum depression (PPD) or postpartum anxiety (PPA). These disorders differ from the “baby blues” in being more severe and longer lasting, often persisting for months and in some cases a year or longer (3, 4). PPD and PPA have a variety of overlapping psychosocial risk factors such as previous or prenatal history of depression and anxiety, as well as stress exposure during pregnancy (5–8). In addition, PPD and PPA can present with similar symptoms, some of which include mood swings, irritability, restlessness, appetite changes, fatigue, and cognitive impairments (4). These consistencies contribute to a high rate of comorbidity of PPD and PPA (9). PPD is distinguished by intense feelings of sadness, hopelessness, and anhedonia or disinterest (4). This disinterest often extends to the baby and can severely impair mother-infant bonding and the ability to adequately provide care (10). PPA can also impact maternal care, though it usually manifests as worry and fear centered around the offspring, leading to behavioral disturbances such as excessive checking on or holding the baby as a neutralization tactic for the compulsive thoughts (11–13). As such, both disorders not only pose significant risk to the mother but to the offspring as well, as early life perturbations in mother-infant interactions are known to have long-lasting negative consequences on development (14–16). More rarely, new mothers may develop Postpartum Psychosis (PP), with a prevalence rate of 1 in every 1,000 births in the general population (17, 18). However, risk for PP is greatly increased for women with a diagnosis of bipolar disorder, schizoaffective disorder or a personal/family history of PP, with up to 50% of individuals in those high-risk categories experiencing an episode after giving birth (19–21). PP, the most severe of postpartum mental illnesses, is accompanied by delusions, hallucinations, paranoia, and detachment from reality (4). PP poses a serious safety risk to both mother and baby, with suicide and infanticide rates at 5 and 4%, respectively (22, 23).

**Table 1:** Summary of prevalence, onset, duration, and symptoms associated with postpartum mental illnesses.

**Disorder**	**Prevalence**	**Onset**	**Duration**	**Symptoms**
Baby blues	80%	Within 2–3 days after giving birth	Up to 2 weeks	Mood swings, Anxiety, Crying, Sleep disturbances
Postpartum depression	10–20%	Longer and more intense than baby blues; May start within a few weeks after giving birth, or later, up until a year after	Months or longer	Mood swings, Anxiety, Crying, Anger, Irritability, Hopelessness, Anhedonia, Trouble concentrating, Thoughts of harming baby, Disinterest/inability to bond with baby, Sleep disturbances, Appetite and weight changes
Postpartum anxiety (generalized, panic disorders, OCD)	10–20%	May start within a few days after giving birth, or later, up until a year after	Months or longer	Panic and fear, Feeling on-edge, Trouble concentrating, Thoughts that are: overwhelming, racing, uncontrollable and illogical, Fear and irrational thoughts centered often around baby, Sleep disturbances, Rapid heartbeat
Postpartum psychosis	Rare; 1:1,000 births	Within 1st week, often within 72 hours, after giving birth	Most severe symptoms last 2–12 weeks, 6–12 months to fully resolve	Mood swings, Hyperactivity, Irritability, Paranoia, Suspiciousness, Hallucinations, Delusions, Difficulty communicating, Sleep disturbances

The etiology of postpartum psychiatric disorders is due, at least in part, to biological factors with most research to date focusing on hormonal and neuronal mechanisms (3, 8, 24–27). However, expanding the scope of research to include interactions with other bodily systems that are known to be influenced by pregnancy is vital for developing new approaches to prevent and treat these conditions. Taking this into consideration, mounting evidence implicates the immune system as a likely candidate with relevance to the onset and maintenance of postpartum mental illnesses. Pregnancy and childbirth are times of immunological change (28) and dysregulated inflammatory responses have been implicated in depression and other psychiatric conditions outside of the peripartum period (29–34). Thus, it stands to reason that a similar mechanism may be involved in peripartum mood disorders. In this review, we will first describe the basic inflammatory changes that occur during the peripartum period, followed by the current state of evidence from both clinical data and animal models that point to aberrant immune function as a likely contributing factor to PPD, PPA, and PP.

## The Immune System During the Peripartum Period

Immune function can generally be defined in terms of innate vs. adaptive responses (35). Innate immune responses are almost immediately mounted in a non-specific fashion against foreign pathogens, whereas the adaptive immune response is slower to emerge but highly targeted to be pathogen specific. In the periphery, natural killer (NK) cells, neutrophils, and monocytes mount the innate response, while T-cells, B-cells, and plasma cells are responsible for adaptive immunity. In addition, subclasses of helper T-cells are critically important for function of cytokines and chemokines, the secreted signaling molecules of the immune system. These subclasses are given the distinction of being Th1 or Th2, each of which have their own associated cytokine profiles. Th1 is considered pro-inflammatory, while Th2 is commonly considered anti-inflammatory. The relative balance of Th1/Th2 factors will either suppress or activate different immune responses and thus depending on this context, will have different consequences for functional biological and behavioral outcomes (36).

Well-defined alterations in these immune responses occur in the periphery during a typical pregnancy (37–42). Throughout the first trimester there is a predominant pro-inflammatory/Th1 bias that is necessary to assist the tissue restructuring and uterine remodeling that occurs during implantation with roles found for interleukins (IL) IL-1β, IL-6, IL-8, IL-17, IL-36, and tumor necrosis factor-α [TNFα] (41, 43–45). A prominent population of NK cells, along with interferon γ (IFN γ) signaling, are also critical in supporting *in utero* maternal angiogenesis during this time (46, 47). By the second trimester, there is a shift to an anti-inflammatory/Th2 profile (i.e., IL-4, IL-10, IL-13, transforming growth factor-β [TGFβ]), which functions to prevent an attack on the semi-allogeneic fetus, allowing for a stage of rapid fetal growth. This immunotolerance is additionally supported by an increase in another subclass of T-cells known as regulatory T-cells (T_reg_ cells). T_reg_ cells suppress the immune response (48, 49), and along with the Th2 cytokines, permit the pregnancy to be carried successfully to term. Alternatively activated macrophages also exert anti-inflammatory capabilities during pregnancy to lend further support for immune suppression during this time (50). Near the time of labor, the immune milieu rapidly returns to a pro-inflammatory state promoting uterine contractions, parturition, and placental expulsion.

The postpartum period is also accompanied by immune changes. For example, plasma markers associated with TNF-ligand families and chemokine ligand 11 (CCL11/Eotaxin) have been shown to be significantly upregulated immediately postpartum (51). The increase in pro-inflammatory markers seen in late gestation persists early in the postpartum period within the first month after delivery along with increased activity of cytotoxic NK and helper T cells (52, 53). The early postpartum immune upregulation may be due to the biological stress induced by parturition. Further, cellular immunity, as measured by cytokine production, does not return to “normal”/pre-pregnancy levels until the first 3–4 months postpartum (54), while transient changes in several lymphocyte populations have been shown to occur over the first year following delivery (55).

When there are disruptions to the otherwise characteristic immune balance of pregnancy, poor health outcomes such as placental insufficiency, pre-eclampsia, or loss of the pregnancy can occur (56, 57). Not surprisingly, immune shifts across the peripartum period also have implications for autoimmune conditions (40). For example, in multiple sclerosis (MS), the increased T_reg_ population is thought to underlie symptom amelioration during pregnancy, while the shift from immunosuppression to inflammation around parturition may trigger aggravation of MS symptoms (58–61). Rheumatoid arthritis is another pro-inflammatory and T_reg_-mediated autoimmune disease characterized both by remission of symptoms during late pregnancy and a significant worsening of symptoms after birth (62, 63). A similar profile is seen in pregnant women with other Th1/T_reg_ related autoimmune disorders such as Graves' disease and myasthenia gravis. Pregnant individuals with these conditions experience an initial worsening of symptoms in early pregnancy that is followed by a decrease of symptoms by the second trimester and then a postpartum relapse in relation to the changing inflammatory profile (64, 65). On the other hand, systemic lupus erythematosus, a Th2 related disease, is accompanied by worsening symptoms during pregnancy, heightened risk for miscarriage, pre-eclampsia, and preterm birth, with concurrent increased risk for maternal mortality (66).

Compared to peripheral immune changes, far less is known about what is occurring in the central immune system across pregnancy and the postpartum period. In homeostatic conditions, peripheral lymphocytes and monocytes are largely prevented from infiltrating the central nervous system (CNS) by the blood brain barrier [BBB; (67)]. Despite this, the peripheral immune system communicates extensively with the CNS *via* secreted factors such as cytokines, chemokines, and hormones and/or through peripheral nerve stimulation (68). Meningeal lymphatic vessels provide an additional connection between peripheral and central immunity (69–71). However, the degree to which peripheral immune alterations during the peripartum period are influencing neuroimmune responses remains unknown. Even in the absence of direct peripheral influence, microglia, the primary innate immune cell in the CNS, are able to respond to hormonal signaling, which is majorly modified during the peripartum period. Thus, it is highly likely that microglia are impacted during this time (72–76). The evidence so far from rodent work (77) does in fact support this hypothesis and has shown that late pregnancy (gestation day 21) is accompanied by a significant reduction in microglia number within various limbic brain regions involved in mood and maternal care, including the prefrontal cortex (PFC), basolateral amygdala (BLA), nucleus accumbens (NAcc), and dorsal hippocampus (HPC). These changes persist across all regions into the mid-postpartum period (postpartum day 8) before returning to virgin levels at weaning (postpartum day 21). Other work has similarly found subregion-specific reductions in microglia within the rodent HPC on the day of birth (78) as well as altered microglia morphology in the HPC mid-postpartum on day 8 (79). While the functional consequences of these peripartum microglia alterations remain unclear, there appears to be a central immunosuppressive phenotype that reflects what is occurring in the periphery, albeit with differing timescales.

In addition to microglia, the relative expression of central cytokine levels has time and region-specific effects across the perinatal period: there is a decrease in IL-6 in the PFC and HPC early in gestation (day 11), though the changes in the HPC persist until parturition (80). Other work has reported increases in both IL-6 and IL-10 at 8 days postpartum in the maternal HPC (77). The extent to which these animal findings translate to humans is yet to be established. A limited number of human studies have collected cerebrospinal fluid (CSF) to measure cytokine expression, though this was done at the time of epidural analgesia administration and as such, lack non-pregnant comparison groups (81, 82). Overcoming methodological barriers to form a clear understanding of the maternal neuroimmune environment at the clinical level is needed.

## Postpartum Depression

### Humans

One line of evidence for immune system dysregulation in PPD comes from work examining immune cell status. In an early study by Hucklebridge et al. (83) higher dysphoric mood ratings in parturient women 4 days following birth were associated with lower circulating Th cells, though there was also a weak association showing lower NK cells. More recently, Osborne et al. (84) sought to further define the profile of these T-cell alterations and found that Th1 and Th17 cells were specifically decreased in patients with PPD. These patients had an average onset of symptoms at 7 days postpartum, though blood samples were not collected until approximately 2 months following birth, demonstrating that these peripheral T-cell deficits persist well into the postpartum period.

Other work has examined the link between inflammatory markers and PPD. For example, Maes et al. (85) assessed inflammatory reactivity in the early postpartum period and the relationship to postpartum mood. Along with non-pregnant controls, serum samples were collected from pregnant women the day before delivery and again at 1 and 3 days postpartum (85). In the women whose depressive scores increased postpartum relative to prenatal measures, there was elevated IL-6 and IL-6 receptor (IL6-R) in the serum at all three time points assessed. Though the timeline of this study is more consistent with a “baby blues” phenotype rather than PPD, subsequent work has looked at later postpartum timepoints. In Bränn et al. (86), levels of pro-inflammatory CXCL1, IL-18, and TNF-receptor family proteins were found to be higher in patients with depressive symptoms at 8 weeks postpartum. Notably, these findings did not change when controlling for timing of onset of depressive symptoms which for some women occurred during pregnancy and thus would indicate that there is likely to be persistent inflammation in depressed individuals postpartum. Another study found increased levels of IL-6 and IL-8 along with decreased IL-2 at 8 weeks postpartum, effects that were similarly associated with depressive symptoms (87). It is important to consider that these latter findings were obtained from a population of patients with severe and suicidal PPD (e.g., symptoms that required hospitalization) suggesting that more severe depressive symptoms may be characterized by a different cytokine profile than less severe cases of PPD, as Bränn et al. (86) found no alterations in IL-6 and IL-8.

Some evidence suggests that immune alterations can predict subsequent mood behavior postpartum. Examining the genetic expression of blood samples taken shortly following delivery, Segman et al. (88) reported transcriptional alterations in markers of immune activation, including those associated with the pro-inflammatory chemokine CXCL10 and the TNF-receptor family. These correlated with later development of PPD. At the protein level, Corwin et al. (89) found that elevated peripheral IL-1β 14 days postpartum predicted increased depressive symptoms at 28 days postpartum. IL-6 levels were also measured in this study, but unlike IL-1β, IL-6 levels did not predict depressive symptoms. In contrast to the lack of relationship between postpartum IL-6 and depressive symptoms, Simpson et al. (90) found that lower levels of IL-6 and IL-10 in blood samples collected in the third trimester of pregnancy were associated with increased depressive symptoms 12 weeks postpartum. Individuals with and without depressive symptoms postpartum showed no differences in third-trimester levels of TNF-α or C-reactive protein (CRP) which is often used as a proxy measure of non-specific inflammation. In contrast, Liu et al. (91) showed that in the days following delivery, elevated IL-6, as well as elevated CRP, were predictive of developing PPD within the first 6 months following parturition. These findings are more in line with other depressed populations, such that patients diagnosed with Major Depressive Disorder outside of the postpartum period have been found to have increased levels of peripheral IL-6 (92).

It should be noted that several studies have reported no association between immune function and PPD. Skalkidou et al. (93) did not find any alterations in serum IL-6 collected at 5 days, 6 weeks, and 6 months after delivery that were associated with PPD symptoms. Moreover, Groer et al. (54) did not uncover any link between mood and serum cytokine levels (IL-2, IL-12, IL-10, IFNγ, TNF-α, IL-4, and IL-6) as assessed at various time points from 1 week to 6 months postpartum. In line with this, Osborne et al. (94), despite finding increased IL-6 and CCL3 in the third trimester in those with greater depressive symptoms while pregnant, did not find any persistence of this inflammation postpartum. Whether the inflammation during pregnancy was predictive of later PPD is unclear based on the analyses conducted.

Some of the inconsistencies across studies is likely attributable to variations in methodology including heterogeneity in timepoints, specific assessments of depression (e.g., clinically diagnosed vs. symptomology), immune markers assessed, sample handling prior to measurement, and the specific measurement techniques used. Demographic differences in subject populations examined could be another moderating factor in observed effects (95, 96). For example, when examining race as a demographic factor, differences in serum cytokine levels were not found but greater depressive symptoms correlated with greater production of IL-6, IL-8, and TNF-α following lipopolysaccharide (LPS) stimulation of blood samples from African American, but not White, women at 7–10 weeks postpartum (97). Because the relationship between depressive symptoms and cytokine markers in peripartum women appears to differ between Black and White women, incorporating racial demographic information as a moderator to future studies is critical. Doing so may also begin to address the underlying causes of racial disparities in PPD prevalence with Black women being more than twice as likely to experience PPD as White women (98). Some work highlights the importance of life history, particularly experiences with the stress of racism, as a contributor to inflammatory changes that drive risk for negative perinatal outcomes, but this has not been well investigated for PPD (99, 100). In one study, higher levels of the inflammatory marker IL-6 were documented in Black women during pregnancy and postpartum (95), although this same study showed neither IL-6 nor TNF-α were associated with depressive symptoms. Other work by Accortt et al. (101) examined pregnant African American women, investigating vitamin D and immune factors during pregnancy as mediators of postpartum depressive symptomology. Their results show that when lower in vitamin D and higher in pro-inflammatory cytokine levels, specifically IL-6 and the relative expression of IL-6 compared to IL-10, self-reported symptoms of PPD were higher. Given the limited number of studies to date, further exploration of the role of inflammation in racial, as well as ethnic, disparities in PPD is warranted (98).

Examining the relative balance or the ratio of both pro-inflammatory and anti-inflammatory factors has gained traction in the literature beyond the single study by Accortt et al. (101) described above. For example, Corwin et al. (102) examined blood cytokine levels during late gestation (third trimester), and again postpartum on days 7 and 14, as well as 1, 2, 3, and 6 months following delivery. Their results show that a significant predictor of developing PPD symptoms was the ratio of IL-8 to IL-10 on postpartum day 14. Furthermore, Bränn et al. (103), when examining 92 markers of inflammation with a multiplex assay from plasma samples collected during late pregnancy (7–23 days before delivery), found that 40 different inflammatory markers were lower in those reporting PPD symptoms at 6–8 weeks postpartum compared with controls. This downregulation, which included decreased IL-8, IL-10, and IL-17, was universal regardless of whether the marker was pro- or anti-inflammatory. These studies indicate that any disruption in the typical cytokine milieu regardless of the direction of the shift, along with the relative pro- vs. anti-inflammatory tone, could be important in mediating later PPD onset. Additionally, the ability of postpartum immune alterations to impact PPD appears to be dynamic and transient, such that certain timepoints may be more influential than others/confer more vulnerability.

All of the studies discussed thus far assessed peripheral inflammatory markers. There are only two studies to date that have looked at the possibility of central cytokine alterations, which may better reflect the inflammatory milieu that the CNS is exposed to during the peripartum period. In a study by Boufidou et al. (81), plasma samples were collected during early labor and cerebrospinal fluid (CSF) samples collected before epidural analgesia was given. In the CSF, positive associations were found between both TNF-α and IL-6 and severity of depressive symptoms, while only plasma TNF-α was positively correlated with depressive outcomes. This work highlights central immune activation in PPD and suggests that neuroimmune changes might not be consistent with what is occurring peripherally. This was further corroborated in a study by Miller et al. (82) which found no correlation between peripheral cytokine levels and cytokine levels in the CSF of pregnant women at the time of delivery. Notably, despite no peripheral alterations in cytokines, Miller et al. (82) did find increased CSF expression of IL-1β, IL-23, and IL-33 and associations with increased prenatal depressive symptoms. Postpartum time points were not examined to determine whether these central alterations persisted or whether they were predictive of later mood disorders. It should be noted, however, that even if peripheral cytokines do not correlate directly with central cytokine levels, this does not diminish the value of peripheral cytokines as potential biomarkers for peripartum mood disorder risk or lessen potential insights into disorder pathophysiology, it simply means that the picture is complex.

The increased capability of sequencing technology in recent years has also been able to provide valuable insight beyond cytokine expression at the gene expression and protein levels as measured in most other studies. A recent genome-wide association study (104) sought to identify genetic markers linked to PPD *via* RNA sequencing of whole blood samples. They found that immune biomarkers, such as genes responsible for encoding TNF-family receptors as well as A disintegrin and metalloproteinase with thrombospondin motifs (ADAMTs), were significantly upregulated in women showing depressive symptoms 2 months postpartum. Further, increased matrix metalloproteinase-8 (MMP8) during late pregnancy was found to be predictive of PPD onset. In addition to strengthening previous evidence pointing to peripheral TNF alterations, imbalances in MMPs and the ADAMTs subfamily of those proteins could be an interesting avenue for future work examining immune-mediated restructuring of the interstitial matrix as well as BBB integrity, both of which are known to be targets for these molecules (105–107), hold relevance to psychiatric symptoms (108, 109), and could point to a potential mechanism of central infiltration of immune cells from the periphery.

### Animals

Recapitulating postpartum mental illness phenotypes in animal models provides valuable insights and furthers our understanding of these complex disorders in a way that cannot be accomplished at the clinical level. Readers are referred to comprehensive reviews on the topic by Perani and Slattery (110) and Li and Chou (111). In brief, two rodent models that have been used to investigate immune and neuroimmune underpinnings of PPD are stress exposure during pregnancy (i.e., gestational stress) and hormone-simulated pregnancy. Though the specific stress paradigms implemented varies across studies, gestational stress models provide construct validity as stress exposure during human pregnancy is a significant risk factor in later experiencing PPD (112, 113). In hormone-simulated pregnancy models, typically conducted in ovariectomized females, exogenous estrogen and progesterone are administered at high doses for several weeks to mimic the sustained elevations in these hormones experienced during normal rodent pregnancy, after which hormone administration is withdrawn to result in abrupt decreases in hormone levels seen in postpartum females at parturition. In addition to the relevant risk factors of stress reactivity and hormonal fluctuations, these models also provide face validity as animals will display behavioral phenotypes similar to what is seen in human PPD, such as increased behavioral despair, anhedonia, and critically, impairments in maternal care (110, 111).

Only one study, to our knowledge, has used a hormone-simulated pregnancy model to explore immune contributions to PPD by examining T cell alterations in peripheral blood samples (114). This study found that after hormone withdrawal, depressive-like behavior increased and this was accompanied by a shift from immune activation to immune suppression as indicated by relative expression of Th cells. Interestingly, both the behavioral and immune effects were reversed by treatment with the antidepressant, fluoxetine, as well as with the herbal medicine Shen-Qi-Jie-Yu-Fang. Overall, this study points to increased inflammatory profiles as underlying postpartum depressive-like behavior mediated by endocrine influences.

More frequently, stress models have been employed to investigate immune changes underlying PPD. O'Mahony et al. (115) subjected pregnant rats to restraint stress from gestational days 14–21. Blood and brain samples were taken at one month postpartum. Increases in blood levels of IL-1β, TNF-α, and IL-10 were found in stressed animals, though no changes in PFC, HPC, or hypothalamus were detected. In other work, Poscillo and Schwarz (78) used a sub-chronic stress model in which pregnant rats were subjected to forced swim from gestational days 16–22. They did not find behavioral differences that were dependent on stress exposure, though their postpartum rats did show a depressive-like phenotype. In these same animals, they detected alterations in PFC and HPC cytokine levels, such that there was increased IL-6 and decreased IL-1β in the PFC and increased IL-4 in the HPC. In a follow up study (116), it was found that central administration of an IL-6R antagonist to unstressed postpartum females attenuated anhedonia as measured in the sucrose preference test, suggesting that central inflammatory signaling is involved in mediating the anhedonic component of depressive-like behavior in mothers. Finally, Tan et al. (117) examined the role of inflammation in PPD and the response to antidepressants. Unpredictable chronic mild stress (UCMS) was applied from gestational days 7–16 to control mice (i.e., C57 wildtype) and microglia-specific autophagy deficient mice as autophagy is a cellular process that can modulate the inflammatory response. Mice were then treated postpartum with the antidepressant, fluoxetine, beginning one week following delivery. Results showed that fluoxetine ameliorated stress-induced depressive-like behavior, but only partially in autophagy deficient mice. Further, it was found that BDNF, autophagy-associated proteins, and IL-10 were upregulated in the HPC of fluoxetine-treated control mice exposed to UCMS who also showed a reduction in inflammatory factors. In contrast, autophagy-deficient mice displayed diminished autophagy, greater inflammation, and reduced BDNF expression when compared with control mice. Given that reduced BDNF expression has also been reported in women with PPD (118), this study suggests that inflammatory processes might be regulating this change, while also providing evidence that fluoxetine may mediate its antidepressant effect in PPD by influencing the inflammatory response.

Preliminary evidence from our lab also implicates altered microglia activity in PPD as we have found increased expression of the phagocytic markers ITGAM and C1q in the PFC at gestational day 21 following UCMS occurring from gestational days 7–20 (119). Moreover, in our model we found increased expression of IL-1β and IFNγ in the PFC at gestational day 21, while there were no differences in peripheral cytokines at this time point or any changes in samples taken at postpartum day 8 (119). Along with previous work from our lab that has shown reduced dendritic spines in the postpartum PFC following gestational stress (120), and other evidence implicating microglia in engulfment of synaptic elements (121–123), these studies suggest that central inflammation and microglia-mediated synaptic remodeling may be resulting from gestational stress. Further exploration of the connections between central immune and neural changes would thus be critical in understanding how stress exposure during pregnancy is contributing to PPD-like behavioral phenotypes.

## Postpartum Anxiety

### Humans

Despite evidence suggesting immune links to anxiety outside of the peripartum period (124–126), relatively little work has examined how immune factors relate to PPA. This may be at least in part because PPA in generally not well studied and because most perinatal immune studies focus on depression.

Maes et al. (85) were the first to investigate an association between postpartum anxiety and peripheral immune markers. They found that parturient women with greater anxiety scores had lower leukemia inhibitory factor receptor (LIFR) prenatally (1 day before delivery) as well as increased IL-6 and IL-1 receptor antagonist postpartum (1 and 3 days after delivery). The lower prepartum anti-inflammatory capacity could distinguish the onset of PPA from that of PPD since, as previously discussed, this same study found that there was an increase in IL-6R at the late stages of pregnancy and no differences in LIFR in women with higher depression scores. The short time frame of this study leaves the remaining questions of whether these immune alterations persist into the postpartum period and whether on their own they are sufficient in giving rise to postpartum mental illness beyond the state measures assessed here.

A more recent study by Osborne et al. (94) also studied possible immune system dysregulation in PPA and the extent to which these might change over time. A broad range of cytokines were examined at 5 timepoints across the peripartum period, including once during each trimester and at 6 and 24 weeks postpartum. During the first and third trimester, both blood IL-6 and CCL3 were significantly higher in those who were anxious than those who were not. Significant differences were not detected at any of the postpartum time points. However, given that antenatal anxiety is a strong predictor of subsequently developing PPA, it would be of interest to know whether any of these immune changes were predictive of later developing anxiety, but this was not examined.

### Animals

The rodent postpartum period is characterized by a reduction in anxiety-like behavior (127). This altered emotionality is thought to be adaptive and necessary for providing proper maternal care (127, 128). Manipulations (i.e., certain gestational stress paradigms, maternal separation) which disrupt typical anxiety-like and fear responses during this period and concurrently impact maternal behavior, may provide insights into PPA. However, distinguishing between depressive- and anxiety-like phenotypes remains a challenge, as manipulations which induce anxiety-like behavior in rodents also often affect depressive-like behavior.

Little work in rodent models has examined immune function as an etiological factor contributing to PPA. A study conducted by Wallace et al. (129) explored the relationship between prenatal inflammation and the development of PPA-like behavior, although the primary focus of the study was on hypertension. Using a rat model of severe preeclampsia that produced upregulated splenic CD4^+^ and CD8^+^ T-cells, they assessed both depressive- and anxiety-like behavior between 2 and 4 weeks postpartum as well as BBB integrity at postpartum week 6. Rats with severe preeclampsia displayed greater anxiety-like behavior as demonstrated by significantly more time spent in the closed arms of an elevated plus maze compared to healthy postpartum controls and more marble burying in the marble burying task. Anhedonia, a component of depressive-like behavior as measured with a sucrose preference test, was not impacted. The results of this study also show that T-cell suppression during pregnancy prevented the PPA-related behaviors as well as the increased BBB permeability that occurred. Whether these mechanisms are translatable to onset of PPA in the absence of preeclampsia remains to be investigated. Nonetheless, as clinical data does point to positive associations between preeclampsia and PPA, this model could also provide insights into any causality underlying this relationship that is currently lacking in human data.

## Postpartum Psychosis

### Humans

A role for immune dysregulation in PP is supported by evidence showing that women who experience PP have higher rates of other complications related to immune dysfunction during their pregnancy (130). For example, immune imbalance occurs in preeclampsia and preeclampsia is a significant risk factor in later developing first onset PP (131). Similarly, other work has found that ~20% of those with PP also had autoimmune thyroid disorder compared to 5% in postpartum controls (132). Interestingly, anti-N-methyl-D-aspartate (NMDA) receptor encephalitis is an autoimmune disorder that has been associated with onset of psychosis symptoms (133, 134). Thus, Bergink et al. (135) sought to investigate whether undiagnosed autoimmune encephalitis may be occurring in patients with PP by conducting an immunohistochemistry-based screening for antibodies. Results from this study showed that in a large cohort of patients with PP, a subset (~4%) had anti-NMDA receptor antibodies suggesting this mechanism might hold relevance to at least some cases of PP.

A growing literature points to links between immune alterations and the development of PP in otherwise healthy mothers (130). One study assessed circulating immune cell types in a population of women who were experiencing the first onset of PP, along with healthy postpartum participants and non-pregnant age-matched healthy controls (53). Those who had PP were reported to have significantly decreased amounts of circulating lymphocytes relative to healthy postpartum participants. Concurrently, those with PP had significantly higher percentages of circulating blood monocytes compared to both healthy postpartum and non-pregnant control participants (53). Another group (136) sought to immunophenotype cells associated with first-onset PP. While there was not a difference associated with PP in overall T_reg_ cells or naïve T_reg_ cells, there was an increase in memory T_reg_ cells compared to healthy postpartum controls. In addition, women with PP had decreased cytotoxic NK cells and elevated regulatory NK cells. Together, these two studies point to altered peripheral immune cells associated with PP, although looking beyond broad classes of cell types (such as distinguishing between cytotoxic vs. regulatory NK cells) will likely provide greater insight into disease etiology.

Another line of work has aimed to establish the profile of circulating cytokines in women with PP. An examination of IL-6 levels by Sathyanarayanan et al. (137) found no difference in women with first onset PP as compared with healthy postpartum women. This finding was confirmed in another study which showed no difference in IL-6 levels of women with PP, those at risk (because of bipolar disorder, schizoaffective disorder or a history of PP), and a group of healthy postpartum controls (138). However, there are data showing higher peripheral serum IL-8 (137) and elevated high sensitivity CRP (hsCRP) in PP as compared to healthy postpartum women (138). In addition to IL-6, IL-8 and CRP, CCL2 (also known as monocyte chemoattractant protein [MCP]-1) has been examined as a possible marker for PP. Although one study found evidence for increased expression of CCL2 in women with first-onset PP (53), this finding was not replicated (137, 138) and thus further investigation is needed. Interestingly, when examining a population that included those with a prior history of PP, BPD, or schizophrenia rather than first onset, Hazelgrove et al. (139) found that inflammatory markers (IL-1β, IL-6, IL-8, TNFα or hsCRP) measured during the third trimester of pregnancy were not predictive of an additional PP episode. Thus, while evidence suggests that dysregulated immune function is associated with PP, more work is needed to parse out the exact role of altered cytokine profiles and how they may differ across individuals with varying predispositions to the disorder.

Case studies of obstetric patients during the COVID-19 pandemic also point toward altered immunity contributing to the onset of PP. Indeed, there is evidence suggesting a possible link between PP and SARS-Cov-2 infection (140, 141). Although in some women who developed COVID-related PP there was a personal or family history of psychiatric illness, this was not universal. Critically, none of the case reports took direct measures of immune activity but increased pro-inflammatory cytokines, including IL-6, TNFα, IL-8, and IL-10, have been observed in the plasma of those with severe COVID-19 infections (142). The additional stress and social isolation of the pandemic when occurring during an already vulnerable period could also be influential environmental triggers despite the varying degrees of predisposition mentioned here. Further exploration of PP in the context of two-hit models (immune perturbation plus environmental stressor) is likely to be a fruitful approach for determining precise etiology of the disorder.

### Animals

The use of animal models to investigate immune underpinnings of PP is almost completely unexplored. Largely, this can be attributed to the lack of well-validated preclinical models of PP. One of two studies that have attempted to model PP in animals was conducted in 2007 by Quilter et al. (143) who profiled genetic expression in a porcine model and found distinct signatures in the hypothalamus of mothers that committed infanticide compared to those who did not. Moreover, cross-species analysis with human tissue showed promise for several candidate genes. The pig provides many advantages to immunology research due to its similarity to the human immune system (144) and thus additional work in a porcine model could have potential to advance understanding of role of the immune system in PP.

Another more recent study (145) which could lend mechanistic insight to the development of PP employed a pharmacological rodent model involving inhibition of steroid sulfatase (SS) in maternal mice. SS is an enzyme that is responsible for the conversion of dehydroepiandrosterone (DHEA) from its sulfated form (DHEAS). Decreased DHEA has been associated with increased pro-inflammatory cytokine expression in the postpartum period (146) while PP and DHEAS levels are positively correlated (147). Inhibition of the SS enzyme in maternal mice increased anxiety-like behavior in an elevated plus maze and resulted in sensorimotor gating deficits as indicated by a decrease in the startle response; both of these behavioral abnormalities were alleviated by treatment with an antipsychotic (145). Reductions in the startle response have been implicated in other neuropsychiatric disorders that serve as the biggest risk factors for developing PP, namely schizophrenia and BPD (148). In rodent models, attenuated startle responsiveness has been found to be partially mediated by peripheral IL-1β signaling (149), but whether IL-1β is involved in the reduced startle response of maternal rodents after SS inhibition was not examined. Indeed, immune measures were not the primary focus of the study although expression of three immune-related genes (CCL2, CXCL1, and IL-33) were assessed and a significant increase in CCL2 following SS inhibition was reported (145). It should be noted that although SS deficiency has been hypothesized to be a risk factor for PP (150), experimental evidence to date is lacking (151, 152). Moreover, as noted above, the results in the human literature regarding CCL2 levels in PP also appear contradictory (53, 137, 138). Thus, possible links among SS, CCL2, and PP onset require further study. Overall, further validation and development of novel preclinical models would be a valuable step toward advancing immune-centered approaches and translatability in PP.

## Immune-Hormone Crosstalk

Though we have considered the immune system in isolation, there are extensive interactions of the immune system with other systems, including the endocrine system. A number of endocrine factors are known to change postpartum including ovarian hormones (24), oxytocin (153), and hormones of the HPA axis (154). Each of these have also been implicated in postpartum mood disorders (6, 8, 155, 156) but the extent to which this is due to hormone-immune crosstalk remains largely unknown. However, emerging evidence is beginning to shed light on this issue. For example, Corwin et al. (102) examined cortisol as a possible mediator of inflammation and PPD and found that increased plasma cortisol was associated with increased levels of IL-6 and IL-10 in depressed mothers at 14 days postpartum (102). However, other work (157) has reported both lower salivary cortisol and lower IFNγ levels in depressed mothers at 4–6 weeks postpartum. Though it is unclear if this could be attributed to the differing time points at which samples were collected, these studies show that dysfunction in other systems like the HPA axis occur alongside alterations in immune signaling and could be facilitating the development and/or persistence of mood dysregulation over the postpartum period.

## Conclusion

Although there is a long standing appreciation that pregnancy-related immune changes are necessary for maternal physical health, there is a more recent growing recognition that the immune system also contributes to maternal mental health. Indeed, the existing evidence points to peripheral immune aberrations in postpartum mental illnesses including PPD, PPA, and PP. Though there is some consistency in immune-related changes across studies, contradictory results also exist and thus a better understanding of the factors responsible for the disparate findings is needed. A critical direction for future work will also be to determine if there are any immune changes that distinguish PPD, PPA and PP from one another as well as whether there are aspects of maternal immune dysregulation that are distinct from those occurring in the context of a diagnosis of depression, anxiety, or BPD outside of the postpartum period. Finally, all the data to date are correlational and thus studies, either longitudinal studies in humans or experiments in animal models, are needed to determine whether immune alterations are driving the development of maternal mental illness or whether they are a result of the disorder itself. A better understanding of immune changes in PPD, PPA, and PP is likely to have implications not only for mothers but also for the offspring as effector molecules such as cytokines are present in breast milk (158, 159). Emerging work linking maternal inflammation to alterations in the microbiota-gut-brain axis and offspring outcomes (160–162) also highlights the critical importance of studying immune dysregulation in postpartum psychiatric conditions.

It should be mentioned that there is another body of literature [reviewed in (56, 163)] describing immune contributions to prenatal episodes of depression and anxiety (94, 164–167). Prenatal history of anxiety and depressive episodes are risk factors for PPD and PPA (4, 6). Thus, immune system dysfunction contributing to prenatal onset could predict later emergence of postpartum disorders, although many studies fail to consider time of onset. This is critical as there is evidence that, at least for PPD, depressive symptoms beginning during pregnancy may be a distinguishable subtype from those that manifest postnatally such that there are differences in their symptomology and severity (168, 169). Further, some subtypes of PPD are more likely to have comorbid anxiety (168, 169). Given this heterogeneity, not all may present an inflammatory profile as is the case in depression outside of the perinatal period (170). Thus, taking different subtypes into consideration will be important in future immune studies and may help to explain some of the current discrepancies in the literature. A similar rationale may apply to PP but further research is needed to identify possible distinct PP subtypes, perhaps based on the presence of previous psychiatric illness or timing of psychosis onset (e.g., immediately postpartum vs. onset after 4-6 weeks).

This review has focused largely on peripheral immune changes primarily because the data on central immune function in postpartum mental illness to date are limited, other than a few studies using PPD animal models or cytokine measures in CSF of women with PPD. Harnessing newer technologies, such as PET imaging of neuroinflammation (171), will begin to fill some of the gaps in the human literature while further studies in animal models will provide greater insights by using cellular and molecular approaches that are not readily feasible in humans. Moreover, the extent to which communication between the peripheral and central immune systems, whether through secreted factors, nerve stimulation, or meningeal lymphatic routes, contributes to the onset of postpartum mental illness remains to be addressed. Finally, it will be necessary to understand how altered immune function might contribute different aspects of neuronal remodeling and neural dysregulation that have been identified in clinical and preclinical studies on PPD, PPA, and PP (3, 26, 61, 172). A more holistic approach to investigating postpartum mental illness that incorporates immune, endocrine, neural, and behavioral levels of analysis will provide much needed information relevant to the prevention and treatment of these serious conditions which impact mothers and their children.

## Author Contributions

CD wrote the manuscript. BL and KL edited the manuscript. All authors contributed to the article and approved the submitted version.

## Funding

This work was supported by NIH Grants MH117482-02 to BL and KL and T32NS105864 to CD.

## Conflict of Interest

The authors declare that the research was conducted in the absence of any commercial or financial relationships that could be construed as a potential conflict of interest.

## Publisher's Note

All claims expressed in this article are solely those of the authors and do not necessarily represent those of their affiliated organizations, or those of the publisher, the editors and the reviewers. Any product that may be evaluated in this article, or claim that may be made by its manufacturer, is not guaranteed or endorsed by the publisher.
